# Greening public health: embedding environmental sustainability in research and practice

**DOI:** 10.3389/fpubh.2026.1888689

**Published:** 2026-08-06

**Authors:** Pascal Petit, Nicolas Vuillerme

**Affiliations:** 1 Université Grenoble Alpes, CNRS, Grenoble INP, LIG, SANGRIA, Grenoble, France; 2 Institut Universitaire de France, Paris, France

**Keywords:** carbon footprint, climate change, climate-resilient public health, environmental footprint, environmental sustainability, ethics, green public health, planetary health

## Abstract

The triple planetary crisis (climate change, pollution, and biodiversity loss) is reshaping health conditions worldwide, yet public health itself contributes to the problem through carbon-intensive and polluting activities. What distinguishes public health is that its environmental footprint arises both from activities shared with other scientific disciplines (e.g., laboratory work, computing, and academic travel) and from activities specific to the field, including surveillance systems, mass screening programs, vaccination campaigns, and community-based interventions. In this perspective, we argue that environmental sustainability should be considered a core dimension of research quality and ethical integrity in public health, consistent with the fundamental principle of “do no harm.” Embedding sustainability requires both systemic transformation and individual engagement. This includes integrating environmental criteria into funding, governance, and evaluation frameworks, as well as adopting frugal, low-carbon, low-pollution practices in everyday work. Importantly, public health holds a distinctive lever compared with other disciplines. Its preventive mission has the potential to reduce the emissions-intensive burden associated with downstream curative care. In this sense, investing in prevention is also an investment in decarbonization and lower pollution. While acknowledging the complexity of measuring environmental impacts and the primacy of health benefits, we contend that a more sustainable, climate-resilient, and environmentally responsible public health is not a compromise; it is a more coherent, effective, and credible approach.

## 1 Introduction: the hidden footprint of public health

Climate change is one of the defining challenges of the 21st century (1). Its association with anthropogenic greenhouse gas (GHG) emissions represents one of the most serious threats to human health and global biodiversity (1, 2). However, it does not occur in isolation. Rather, it forms part of a triple planetary crisis, encompassing climate disruption, chemical pollution, and the degradation of biodiversity and natural systems (3, 4). These three crises are deeply interconnected, with overlapping drivers, mechanisms, and consequences. At their core, they are fueled by common socio-economic processes, including the intensification of resource extraction, unsustainable production and consumption patterns, and persistent structural inequalities. Their effects are not only cumulative but also mutually reinforcing. For example, environmental pollutants degrade ecosystems, weakened ecosystems become more vulnerable to climate impacts, and climate change further accelerates both biodiversity loss and the spread of environmental contaminants (5). The loss of biodiversity has far-reaching implications, increasing the risk of emerging infectious diseases, compromising water and food security, and disrupting essential ecosystem functions that underpin human health. In parallel, chemical pollution, defined as the introduction of substances into the environment at rates exceeding their safe assimilation, has been identified as one of the nine planetary boundaries and is now a global threat to ecosystem stability (5, 6). Importantly, these crises rarely occur in isolation. They often interact as compound environmental stressors, producing cascading effects that are more severe and complex than any single crisis alone, with disproportionately high impacts on marginalized populations (3). Collectively, the triple planetary crisis affects almost every health determinant, including food and water security, infectious disease patterns, and mental health (1, 2, 7, 8). In response, the World Health Organization has called for the integration of mitigation and adaptation strategies across all domains of health governance (1).

Academic research itself contributes to the triple planetary crisis (2, 9–13). Individual researchers can generate 2–37 tons of CO2 equivalents (CO2e) yearly, with high heterogeneity across disciplines (e.g., 18–37 tons for astronomy or 4–15 tons for life sciences) and seniority (e.g., 9.5–12 tons for a senior, 4 tons for a postdoc, or 2 tons for a PhD candidate). These levels exceed the annual 1.5 tons per capita target compatible with the Paris Agreement (9). Scientific activities rely on carbon-intensive and polluting infrastructures, including laboratory operations, procurement of materials, international travel, and digital computing (2, 9–13). Laboratories, for instance, consume five times more energy per unit area than standard office spaces and account for 60–65% of the total energy consumption of a university (9, 14). Manufacturers rarely disclose information on the environmental footprint of laboratory supplies and equipment, making it difficult to make informed purchase decisions. Single-use items (e.g., gloves, syringes) contribute substantially to the environmental impact of laboratories through embodied carbon and waste (15). Academic travel is another major contributor, with conference participation accounting for up to 35% of a researcher’s annual carbon footprint. A single large scientific meeting can produce tens of thousands of metric tons of CO2e (9, 16). Research activities are also increasingly dependent on large-scale computational resources. The digital sector is estimated to contribute 2.1–3.9% of global GHG emissions, and health is the fastest-growing sector in the global datasphere (2, 17). Emissions vary widely depending on computational tasks, hardware, software efficiency, and the carbon intensity of the local electricity grid (16, 18). Memory over-allocation, unnecessary parallelization, and redundant computation further increase energy consumption (16). In addition, the extraction of raw materials for digital technologies and the informal recycling of e-waste in low-income countries create disproportionate environmental and health burdens, raising important equity concerns (17).

These issues are common to many scientific and medical disciplines. However, what makes the environmental footprint of public health distinctive is its extent and diversity of activities. Some activities, such as laboratory work, computing, data infrastructure, procurement, travel, and study designs, overlap with other disciplines. For example, experimental research raises questions about the 3Rs framework (replacement, reduction, and refinement) not only as an ethical principle but increasingly as an environmental one (9). A single clinical trial generates around 180 tons of CO2e annually, and clinical research overall has been estimated to produce around 100 million tons of CO2e per year, comparable to the emissions of one of the world’s top 40 emitting countries (9). Similarly, cohort studies require substantial resources, including sample collection, storage infrastructure (e.g., biobanks), and ongoing data management (16, 19). In addition, while the growing integration of digital technologies into healthcare offers genuine population health benefits, it also increases data storage and processing demands, with associated environmental costs (17, 20). Public health also encompasses activities that are largely unique to the discipline, such as surveillance systems, screening programs, vaccination campaigns, health promotion, community interventions, and the large-scale translation of evidence into policy. Each of these activities has its own environmental footprint, but, to our knowledge, none has been systematically characterized.

Available estimates of public health’s environmental impact rely on proxies derived from the broader healthcare sector. Public health activities accounted for approximately 5.9% of total national healthcare-related GHG emissions between 2002 and 2013 in the US (21), 5.8% in 2015 in Canada (22), 6% in 2015 in Australia (23), and 4% in China in 2012 (24). These figures are informative but incomplete. They are based on different methodologies and fail to capture the full spectrum of public health activities. In addition, existing estimates predate the COVID-19 pandemic, which led to a substantial increase of public health activities worldwide, including mass vaccination campaigns, large-scale testing, and digital surveillance, each carrying important environmental costs. As pandemics and infectious disease outbreaks are expected to become more frequent, the environmental footprint of public health is likely to increase, making its systematic measurement increasingly urgent.

The broader health sector contributes around 5% of global environmental impacts (2, 8, 25), corresponding to an estimated loss of 3 million disability-adjusted life-years (DALYs) annually (25). Supply chains account for approximately 71% of these emissions, compared with 12% from energy use (26). This pattern likely extends to public health, where supply chains include reagents, single-use plastics, cold-storage systems, digital technologies, and field logistics. The environmental footprint of healthcare extends well beyond GHGs. In the US, the healthcare sector was responsible in 2013 for around 12% of acid rain-related emissions, 10% of smog formation, and 9% of air pollutants, with the resulting pollution that was estimated to cause nearly 470,000 DALYs lost annually (21). In Canada, healthcare activities generated over 200,000 tons of non-carbon pollutants in 2015, causing an estimated 23,000 DALYs lost from direct exposure to hazardous substances, with public health activities alone contributing several hundred DALYs through mainly particulate matter and carcinogen exposure (22). Globally, healthcare accounted for an estimated 2.8% of particulate matter emissions, 3.4% of nitrogen oxides, and 3.6% of sulfur dioxide, and all these indicators increased between 2000 and 2015 (27). In 2021, air pollution from health system operations and supply chains was estimated to have caused 4.6 million DALYs globally, a figure nearly 20% higher than in 2020 (28). Beyond air pollution, analysis of the Dutch healthcare sector revealed that pharmaceuticals and chemical products were the largest contributors across multiple environmental impact categories, including material extraction (13% of the national footprint), water consumption (7.5%), and land use (7.2%), with most of these impacts occurring outside national borders (29). Pharmaceutical contamination of aquatic ecosystems, through the discharge of drugs that are biologically persistent, threatens freshwater biodiversity, contributes to antimicrobial resistance, and may undermine progress toward sustainable development goals (6). The use of nanomaterials in drugs and vaccines introduces further uncertain risks to both human health and ecosystems (4). Despite this broad footprint, available estimates of healthcare’s environmental impact remain almost exclusively focused on carbon emissions, with chemical pollution, biodiversity loss, water use, and waste generation largely unmeasured (21, 22, 27, 29).

The relative invisibility of public health’s environmental footprint is problematic. As scientists increasingly engage in public discussions on planetary crisis, their credibility is scrutinized (11). A field that examines the environmental determinants of health while neglecting its own ecological impact jeopardizes its credibility. The public health community (e.g., researchers, practitioners, policymakers, and educators) has a responsibility to integrate climate accountability and planetary health into both daily practice and institutional structures. This perspective argues that environmental sustainability must be recognized as integral to research quality and professional responsibility in public health. This is not about condemning public health for its emissions, which may represent a small share of total healthcare-related environmental impacts (e.g., GHGs), but about the exemplary role that a prevention-oriented discipline is positioned to play. The goal is not to restrict science or compromise health outcomes but to recalibrate and optimize practices in a way that balances health benefits with environmental costs (30, 31). Societal change is unlikely to occur without leadership from the health sector (30).

## 2 The paradox of healing and harming

Public health occupies a unique position among scientific and medical disciplines, characterized by a two-dimensional paradox. Like other disciplines, it contributes to the environmental degradation it seeks to address. At the same time, its central mission, preventing disease and promoting health, can generate environmental co-benefits that remain underexplored (32). Indeed, a healthcare system that prevents disease reduces reliance on pharmaceuticals, high-emission procedures, and energy-intensive hospital infrastructure (33). Shifting from a curative to a preventive model therefore offers a pathway to both improved health outcomes and a reduced environmental footprint (32, 34–36). In this way, public health is not only responsible for reducing its footprint but also has the potential to lower the environmental burden of the entire healthcare system (32).

**Figure 1:**
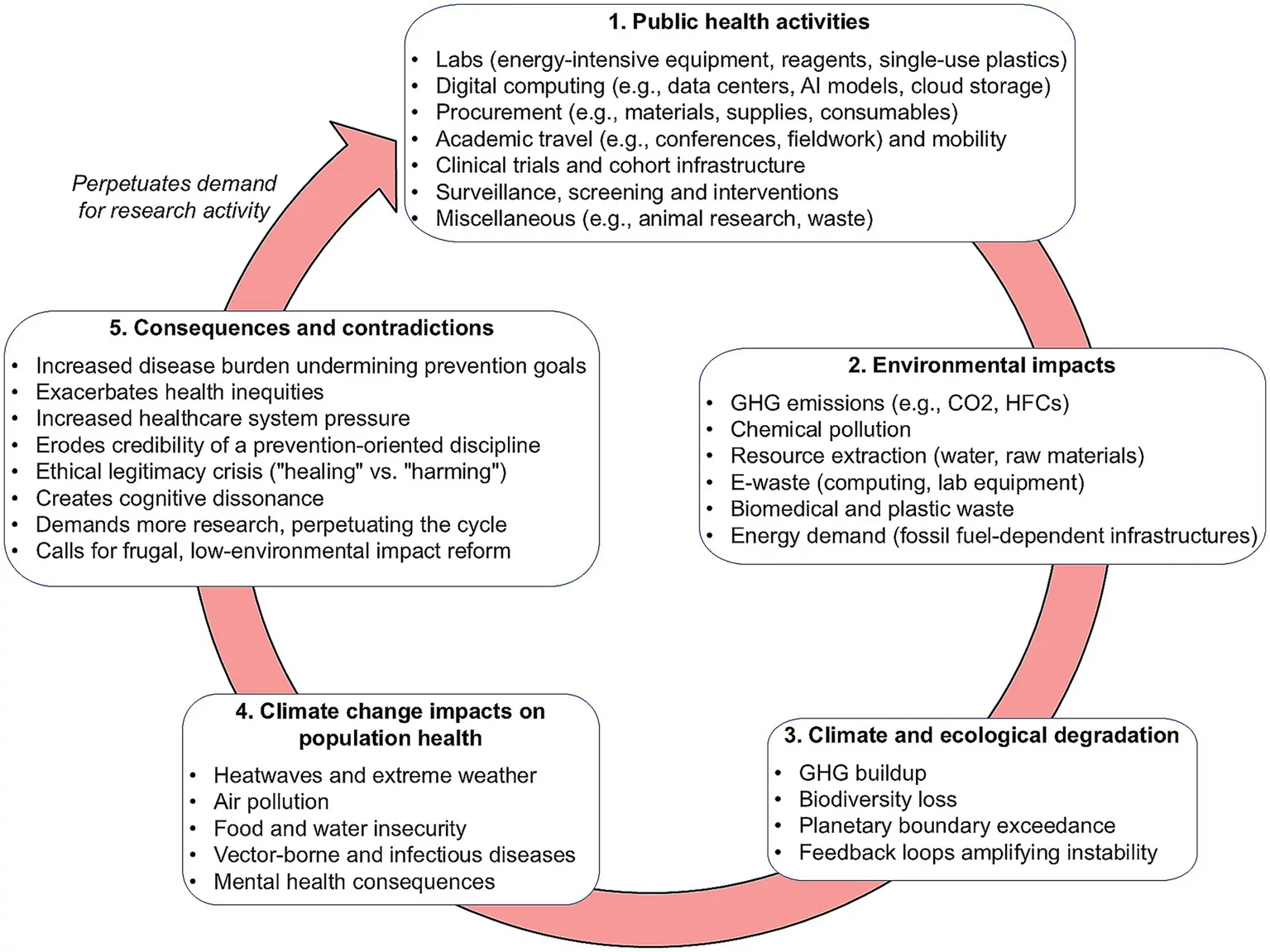
The paradox of public health activities in the Anthropocene: a feedback loop from research activities to environmental impacts, climate and ecological degradation, population health consequences, and scientific and ethical contradiction. AI, artificial intelligence; GHG, greenhouse gas; HFC, hydrofluorocarbons.

Public health infrastructure remains closely tied to fossil fuels, resource-intensive systems, and expanding digital networks that contribute to the triple planetary crisis (Figure 1). Consequently, the discipline risks reinforcing the very crises it aims to mitigate. This contradiction may create a sense of moral tension among professionals who may recognize their environmental impact while feeling constrained by institutional norms that prioritize productivity and visibility over sustainability (12). Public health professionals may experience a cognitive dissonance between their commitment to “do no harm” and the environmental consequences of their work; between career incentives that encourage mobility and output and the need for low-carbon and low-pollution practices; and between the pursuit of precision and the ecological cost of computation (12).

It is important to note that much of the environmental footprint associated with public health arises from systems beyond the direct control of individuals, such as energy grids, global supply chains, and manufactured technologies. Recognizing this fact helps clarify the nature of responsibility. The issue is not individual fault, but professional coherence, aligning the values of a prevention-focused discipline with the realities of how research is conducted. Encouraging individual behavior change without addressing systemic changes is insufficient and may even increase frustration (37). Individual action remains meaningful when supported by collective efforts and fair institutional frameworks.

## 3 Rethinking abundance: toward frugal, low-carbon, and low-pollution public health activities

Achieving environmentally sustainable research requires rethinking how knowledge is produced, considering ecological constraints (31). Frugality should not be confused with austerity. It represents a principle of sufficiency, producing knowledge that is both rigorous and resource-conscious. It involves asking what level of data, computation, and infrastructure is truly necessary to answer a research question (2, 38).

For activities shared with other disciplines, several practical approaches can reduce environmental impact [e.g., (9, 10, 39)]. In computational work, “data temperance” emphasizes collecting only what is necessary (2, 40). This can be supported by optimizing code, improving software efficiency, choosing low-carbon data centers, and using parsimonious models (2, 10, 16). Techniques such as model pruning or early stopping can reduce emissions without compromising validity (2, 41, 42). Open-source tools such as Green Algorithms, CodeCarbon, and eco2AI allow researchers to estimate the environmental impact of their computational work, although they mostly remain limited to machine learning tasks (2, 16, 18, 41, 43, 44). In laboratory settings, reusing materials, improving HVAC and freezer management, and reducing single-use plastics can substantially lower emissions without affecting research quality (15, 45, 46). For example, reusing 50 mL conical tubes can reduce the CO2e emissions by more than elevenfold compared with single-use alternatives (15). Reusing existing datasets and applying FAIR (Findable, Accessible, Interoperable, Reusable) principles also reduces both environmental and financial costs (19). Virtual or hybrid conferences can reduce travel-related emissions by up to 86% (47), while telemedicine has already demonstrated substantial emission savings (48, 49), though these benefits depend on careful implementation to avoid rebound effects (10, 17).

For public health-specific activities, frugality requires tailored approaches. In exposome research, focusing on a smaller set of relevant high-impact exposures can reduce measurement and computational burden while maintaining scientific and clinical relevance (50). Surveillance and screening programs can apply diagnostic stewardship to ensure that data collection aligns with real needs rather than technical possibilities (46). Incorporating life cycle assessment (LCA) into public health interventions can make environmental costs visible alongside health benefits and inform decision-making (51–53). For example, in supervised toothbrushing schemes and in inhaler switching interventions, LCA was used to estimate environmental costs alongside clinical effectiveness and guide policy (51, 52). More broadly, LCA frameworks applied to public health interventions should be designed to capture the full spectrum of environmental impacts, not only carbon emissions but also air and water pollutant releases, pharmaceutical contamination, material extraction, water use, and waste generation, consistent with the multi-dimensional nature of public health’s ecological footprint (4, 21, 22, 27, 29). Adopting a patient pathway perspective can also help identify environmental hotspots across the continuum of care, enabling more targeted and systemically meaningful interventions to reduce the environmental impact of health services (49).

This approach does not limit scientific ambition. Large-scale collaborative research remains essential. The goal is not to minimize environmental impact at any cost but to find an optimal balance between health benefits and ecological consequences. Strategies such as data sharing, federated analysis, and improved computational efficiency can support both sustainability and scientific excellence.

Although measuring environmental impact remains challenging (49, 54, 55), inaction is no longer justifiable (30, 40). While LCA provides a standardized framework, its application to research activities is limited by data availability (25, 49). Nevertheless, many institutions already hold relevant data (e.g., energy use, procurement, and travel) that can inform practical action (55). Early efforts, even if imperfect or small, can build momentum and have meaningful impact.

## 4 Levers for change: from institutions to everyday practice

**Figure 2:**
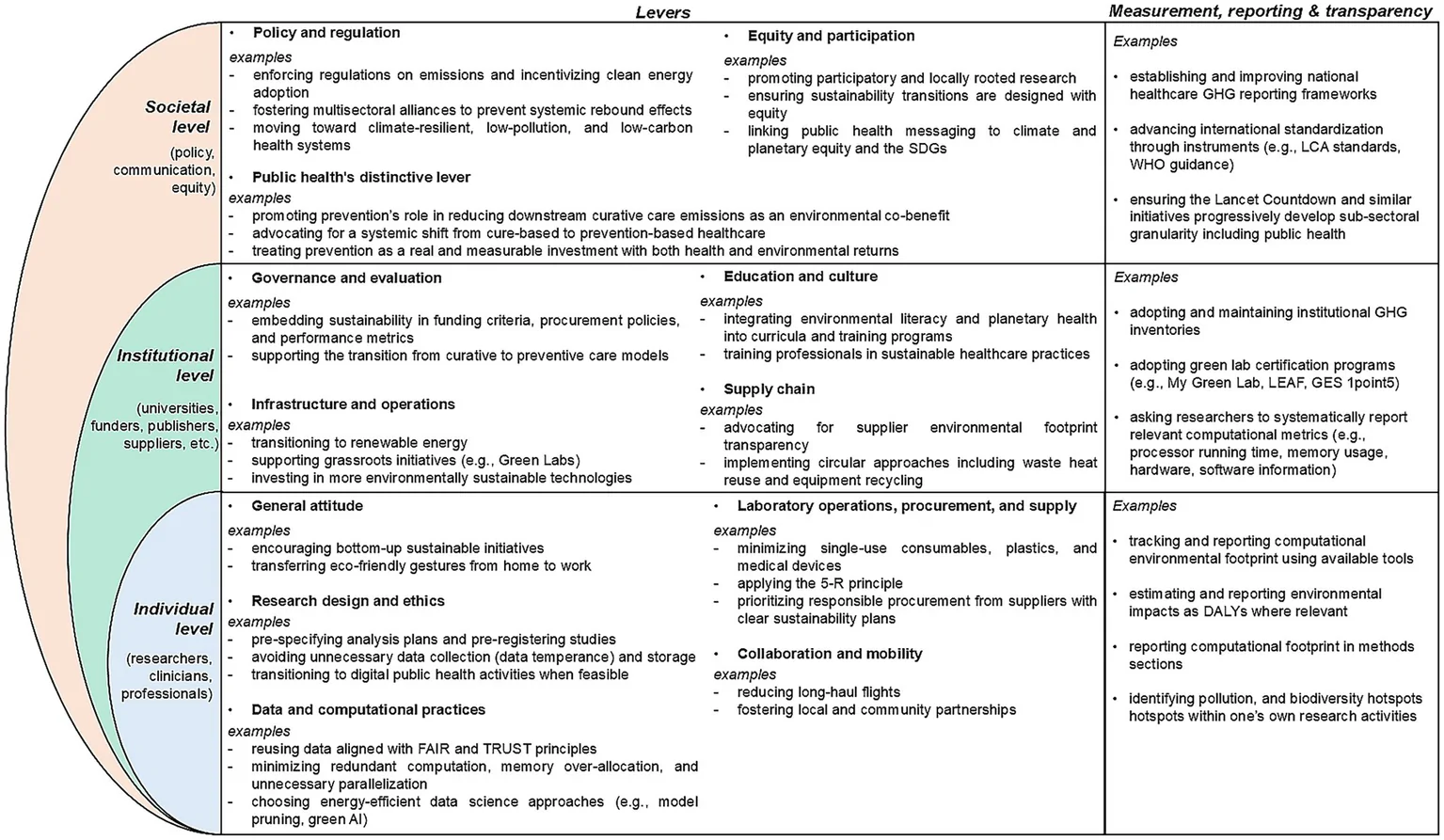
Systems diagram of levers for change in green public health. 5-R, refuse, reduce, reuse, repurpose, recycle; AI, artificial intelligence; DALY, disability-adjusted life-year; FAIR, findable, accessible, interoperable, reusable; GHG, greenhouse gas; LCA, life cycle assessment; LEAF, laboratory efficiency assessment framework; SDG, sustainable development goal; TRUST, transparency, responsibility, user focus, sustainability and technology; WHO, World Health Organization.

Advancing climate-resilient and low-pollution public health requires coordinated action at multiple levels (Figure 2; Supplementary Tables S1–S3). Environmental sustainability cannot rely solely on individual initiatives (9, 37); it must be embedded within governance and institutional systems (8, 31, 37).

At the individual level (Supplementary Table S1), professionals can adopt low-carbon and low-pollution practices in computation, laboratory work, travel, and study design as described above. Locally rooted and participatory research can reduce resource use while enhancing relevance and community engagement. Public health professionals span a wide range of roles, from researchers and clinicians to practitioners and policymakers, and this diversity is itself a strength. Each professional group has its own sphere of influence, and together they are well positioned to act as models and leaders for environmentally sustainable practices within and beyond the health sector.

At the institutional level, funders and universities play a critical role (Supplementary Table S2). Sustainability should be integrated into evaluation criteria, procurement policies, and funding decisions (8, 13, 37, 56). Indicators such as data reuse, emission reduction, and methodological efficiency should be recognized alongside traditional metrics (e.g., performance). Institutions should also invest in renewable energy, sustainable digital infrastructure, and grassroots initiatives such as Green Labs (9, 57, 58). Greater transparency in supply chains would support more informed purchasing decisions, and emerging platforms such as *Lancet MedZero*, designed to provide robust carbon data across clinical and research pathways, may contribute to reducing environmental impacts (59). Scientific publishers could also accelerate change by requiring transparency on environmental impacts in methods sections and supporting low-carbon and low-pollution dissemination formats (37, 57). Integrating environmental impact assessment into health technology assessment frameworks represents another key institutional lever. As decision-makers assess the value of interventions, there is increasing recognition of the need to account for their full environmental consequences, including chemical pollution, biodiversity impacts, and waste generation, alongside clinical and economic outcomes (4, 36). For example, pharmaceutical pollution, antimicrobial resistance, and the environmental risks associated with digital health technologies and nanomaterials require assessment approaches that extend beyond carbon accounting (4, 6). Such frameworks would not only help mitigate adverse effects but also support the identification and prioritization of interventions that generate positive environmental co-benefits. Public health interventions targeting tobacco use, obesity, meat consumption, and air pollution, for instance, have been projected to avoid approximately 1.5 gigatons of CO2e between 2014 and 2050 (36), illustrating that prevention can serve both health and environmental objectives. The credibility of these approaches, however, depends on their methodological rigor (49). Preventing greenwashing requires more than formal commitments; it calls for clearly defined environmental performance standards, independent verification, and priority-setting grounded in measurable, long-term objectives. Without these elements, efforts risk remaining fragmented or limited to incremental improvements that fall short of the scale of the challenge. In this context, complementary forms of knowledge, including practical experience, traditional knowledge, and integrated knowledge systems, can inform decision-making, particularly where the environmental impacts of complex interventions remain difficult to quantify. Recognizing these perspectives may help address measurement gaps and support more coherent choices across interventions. Ultimately, public health should move beyond limiting negative impacts toward actively prioritizing interventions with positive environmental effects. Education is also a decisive lever. Integrating environmental literacy, green computing, and planetary health into public health training would play a key role in promoting environmental accountability and nurturing a culture of ecological responsibility across the discipline (7–9, 11, 12, 56, 60–63). This imperative extends well beyond public health: embedding sustainability into clinical training more broadly, including in surgery, has recently been framed as a population-level intervention with the potential to reduce healthcare-related emissions while building more climate-resilient health systems (62, 63).

At the societal level (Supplementary Table S3), public health communication should emphasize that the triple planetary crisis is fundamentally a health issue and that prevention-based systems are part of the solution (31). Equity and environmental justice must remain central. For example, policies that restrict international travel risk further marginalizing researchers from low- and middle-income countries, who already face structural barriers to network-building and visibility. Digital tools can help mitigate this risk, but only if access is equitable.

## 5 Conclusion: deciding to act

Public health must confront the environmental consequences of its own practices, not because it is the largest contributor, but because its values demand it. A discipline grounded in prevention and committed to protecting present and future generations cannot ignore its role in the triple planetary crisis. Progress requires action at multiple levels. Individuals can adopt more sustainable practices, while institutions and societies must align incentives, structures, and policies to support these efforts. Neither level can succeed alone. Meaningful change depends on their interaction. However, achieving sustained environmental improvement will require more than marginal or incremental adjustments. It depends on structural transformation, meaningful institutional commitment, and accountability mechanisms proportionate to the magnitude of the crisis. The need for environmental coherence is not specific to public health but extends across the entire healthcare sector (49), where awareness of the environmental consequences of clinical and organizational decisions remains largely insufficient. Raising this awareness at all levels (e.g., among clinicians, hospital administrators, health technology assessors, policymakers, and insurers) is a prerequisite for embedding environmental considerations into the decisions that shape how care is delivered, financed, and evaluated (4, 28, 64). Public health, however, by its preventive mandate and its focus on protecting both current and future generations, is particularly well positioned to lead, not merely by reducing its own footprint but by actively designing interventions that generate long-term positive environmental and health co-benefits.

Ultimately, greening public health is not just a technical adjustment; it is a moral imperative aligned with the principle of “do no harm.” Frugality and environmental sustainability are not barriers to discovery; they reinforce its credibility in a world shaped by ecological limits. By strengthening its preventive mission, public health can also reduce the environmental burden of healthcare more broadly, making investment in prevention an investment in decarbonization and lower pollution. In this sense, a greener, more sustainable, climate-resilient, and low-pollution public health system is not a compromise; it is a more coherent, more effective, and more credible path forward.

## Data Availability

The original contributions presented in the study are included in the article/Supplementary material, further inquiries can be directed to the corresponding author.
