# Supplementary material for: Greening public health: embedding environmental sustainability in research and practice
Source: Front Public Health. 2026 Aug 6;14:1888689. doi: 10.3389/fpubh.2026.1888689 (PMC13490115; doi:10.3389/fpubh.2026.1888689)
Supplement: Supplementary file 1 [file Table_1.DOCX]

Supplementary Material

# 1 Supplementary Tables

**Table S1**: Levers for change and associated measurement, reporting and transparency in green public health at the individual level.

| **Lever** | **Measurement, reporting and transparency** |
| --- | --- |
| ***General*** - staying informed - being aware of rebound effects - encouraging bottom-up sustainable initiatives - raising awareness - avoiding large email attachments - transferring eco-friendly gestures from home to work  ***Laboratory operations, procurement, and supply*** - prioritizing responsible procurement from suppliers with clear sustainability plans - reducing waste - running equipment (e.g., autoclaves) only when full - replacing toxic chemicals with less toxic alternatives where possible - switching off equipment when not in use - reusing labware and materials where safety permits - optimizing HVAC and freezer management - installing low-flow faucets - sorting waste properly - closing fume hood sashes when not in use - consolidating orders to reduce transport - minimizing single-use consumables, plastics, and medical devices - implementing explicit biomedical and plastic waste management - designing logistically lean field protocols - applying the 5-R principle  ***Research design and ethics*** - pre-specifying analysis plans and pre-registering studies - avoiding unnecessary data collection (temperance) and storage - applying parsimony in exposure selection - incorporating LCA in study and intervention design - applying diagnostic stewardship principles at population level - minimizing animal research when evidence permits (3-R principles) - matching testing and data collection to genuine public health need - designing and evaluating interventions with sustainability criteria - adopting frugal science principles - using virtual study components and remote monitoring - sharing protocols and datasets - replacing outdated techniques - promoting centralization of data centers and biobanks where appropriate - transitioning to digital public health activities when feasible  ***Data and computational practices*** - applying digital temperance - reusing data aligned with FAIR, GREENER and TRUST principles - minimizing redundant computation, memory over-allocation, and unnecessary parallelization - refining simulations and optimizing code efficiency - using parsimonious models with fewer variables - choosing energy-efficient data science approaches (e.g., model pruning, green AI) - selecting energy-efficient data centers - using up-to-date software  ***Collaboration and mobility*** - reducing long-haul flights - prioritizing virtual, hybrid, and local collaboration adopting hub-and-spoke in-person models - taking advantage of remote participation - optimizing travel routes and timing - working collectively - fostering local and community partnerships | - following systematic reporting guidelines [e.g., (1)]  - incorporating environmental impact in ethics review  - conducting cost-benefit and cost-effectiveness analyses that include environmental costs  - estimating and reporting environmental impacts as DALYs where relevant  - conducting lab audits  - reporting computational and environmental impact metrics in methods sections  - tracking and reporting computational carbon footprint using available tools [e.g., (2, 3)]  - making environmental costs visible alongside health outcomes  - identifying carbon hotspots within one’s own research activities  - following standard operating procedures to track energy use and carbon emissions |

*Note*: 3-R: refinement, reduction, and replacement (4); 5-R: refuse, reduce, reuse, repurpose, recycle (5); AI: artificial intelligence; DALY: disability-adjusted life-year; FAIR: Findable, Accessible, Interoperable, Reusable (6); GREENER: Governance, responsibility, estimation, energy and embodied impacts, new collaborations, education, research (7); HVAC:, heating, ventilation, and air conditioning; LCA: life cycle assessment; TRUST: Transparency, Responsibility, User focus, Sustainability and Technology (8).

**Table S2**: Levers for change and associated measurement, reporting and transparency in green public health at the institutional level.

| **Lever** | **Measurement, reporting and transparency** |
| --- | --- |
| **Governance and evaluation** - embedding sustainability in funding criteria, procurement policies, and performance metrics - rewarding data reuse, parsimony, low-carbon methods, and reproducibility as markers of quality - requiring environmental transparency in grant applications and publication methods sections - ensuring that funders expect and eventually require that funds are spent with environmental responsibility - supporting low-carbon dissemination formats - supporting the transition from curative to preventive care models - setting research agendas that encourage sustainable practices - establishing climate action policies at all levels - investing in local or distant carbon offsetting where other reductions are not feasible - enabling conference organizers to offer virtual participation and local hubs  **Infrastructure and operations** - transitioning to renewable energy - investing in sustainable IT infrastructure - supporting grassroots initiatives (e.g., Green Labs) - improving building energy efficiency - investing in more environmentally sustainable technologies - reducing the size and optimizing animal facilities - streamlining digital processes - favoring videoconferencing - using existing platforms to accelerate deployment of climate-responsive solutions in public health settings [e.g., Climate x Health Challenge (9), Lancet MedZero (10)] - designing systems for sharing best practices without increasing bureaucracy - adopting formal environmental management systems  **Education and culture** - integrating environmental literacy and planetary health into curricula and training programs - training in sustainable computing and green data science - training professionals in sustainable healthcare practices - creating a culture of sustainability in labs that is flexible rather than prescriptive  **Supply chain** - sending signals to manufacturers through procurement criteria requiring carbon value transparency, supplier footprint disclosure, and ambitious decarbonization plans - implementing circular approaches including waste heat reuse and equipment recycling | - adopting and maintaining institutional GHG inventories  - adopting green lab certification programs [e.g., My Green Lab, LEAF, GES 1point5 (11, 12)]  - identifying institutional carbon hotspots  - drawing on existing measurement frameworks [e.g., (13)] and energy monitoring tools  - ensuring procurement carbon transparency  - using standardized metrics in funding criteria  - asking researchers to systematically report relevant computational metrics (e.g., processor running time, memory usage, hardware, software information)  - developing conference and journal checklists disclosing energy consumption, computational complexity, and experimental parameters |

*Note*: GHG: greenhouse gas; IT: information technology; LEAF: Laboratory Efficiency Assessment Framework (12).

**Table S3**: Levers for change and associated measurement, reporting and transparency in green public health at the societal level.

| **Lever** | **Measurement, reporting and transparency** |
| --- | --- |
| ***Policy and regulation*** - enforcing regulations on emissions and incentivizing clean energy adoption - enforcing policy and regulation regarding healthcare contributions to climate change - providing green incentives, subsidies, or tax benefits for institutions investing in low-carbon digital technologies and energy-efficient computational infrastructure - integrating environmental performance metrics into procurement criteria and accreditation standards - requiring suppliers to manufacture more sustainable products across their full life cycle - fostering multisectoral alliances to prevent systemic rebound effects - encouraging collaborative public-private funding models to distribute the financial burden of transitioning to greener solutions - moving toward climate-resilient and low-carbon health systems  **Public health's distinctive lever** - promoting prevention’s role in reducing downstream curative care emissions as an environmental co-benefit - advocating for a systemic shift from cure-based to prevention-based healthcare - treating prevention as a real and measurable investment with both health and environmental returns  **Equity and participation** - promoting participatory and locally rooted research - ensuring sustainability transitions are designed with equity - linking public health messaging to climate equity and the SDGs - supporting citizen and community science initiatives | - establishing and improving national healthcare GHG reporting frameworks  - advancing international standardization through instruments (e.g., LCA standards, WHO guidance)  - ensuring the Lancet Countdown and similar initiatives progressively develop sub-sectoral granularity including public health  - supporting the development of a dedicated carbon footprint framework for public health activities that accounts for both shared and discipline-specific emission |

*Note*: GHG: greenhouse gas, LCA: life cycle assessment, SDG: Sustainable Development Goal, WHO: World Health Organization.

# 2 References

1. Henderson P, Hu J, Romoff J, Brunskill E, Jurafsky D, Pineau J. Towards systematic reporting of the energy and carbon footprints of machine learning. *J Mach Learn Res*. (2020) 21:1–43. doi: 10.48550/arXiv.2002.05651.

2. Budennyy SA, Lazarev VD, Zakharenko NN, Korovin AN, Plosskaya OA, Dimitrov DV, et al. eco2AI: Carbon Emissions Tracking of Machine Learning Models as the First Step Towards Sustainable AI. *Dokl Math*. (2022) 106:S118–S128. doi: 10.1134/S1064562422060230.

3. Mansour FR, Nowak PM. Introducing the carbon footprint reduction index (CaFRI) as a software-supported tool for greener laboratories in chemical analysis. *BMC Chem*. (2025) 19:121. doi: 10.1186/s13065-025-01486-2.

4. Critser R, Locke P. How Should the 3 R's Be Revised and Why?. *AMA J Ethics*. (2024) 26:E724–E729. [doi: 10.1001/amajethics.2024.724](https://doi.org/10.1001/amajethics.2024.724).

5. Sharma S, Basu S, Shetti NP, Kamali M, Walvekar P, Aminabhavi TM. Waste-to-energy nexus: A sustainable development. *Environ Pollut*. (2020) 267:115501. [doi: 10.1016/j.envpol.2020.115501](https://doi.org/10.1016/j.envpol.2020.115501).

6. Inau ET, Sack J, Waltemath D, Zeleke AA. Initiatives, Concepts, and Implementation Practices of the Findable, Accessible, Interoperable, and Reusable Data Principles in Health Data Stewardship: Scoping Review. *J Med Internet Res*. (2023) 25:e45013. [doi: 10.2196/45013](https://doi.org/10.2196/45013).

7. Lannelongue L, Aronson HG, Bateman A, Birney E, Caplan T, Juckes M, et al. GREENER principles for environmentally sustainable computational science. *Nat Comput Sci*. (2023) 3:514–521. [doi: 10.1038/s43588-023-00461-y](https://doi.org/10.1038/s43588-023-00461-y).

8. Lin D, Crabtree J, Dillo I, Downs RR, Edmunds R, Giaretta D, et al. The TRUST Principles for digital repositories. *Sci Data*. (2020) 7:144. [doi: 10.1038/s41597-020-0486-7](https://doi.org/10.1038/s41597-020-0486-7).

9. PATH. Climate X Health Challenge. (2024). Available from: <https://media.path.org/documents/ClimateXHealth_final_brochure.pdf>. (Accessed 19 May, 2026).

10. Watts N, Andrew T, Bagenal J, Eckelman MJ, Fry J, Henson A, et al. The Lancet MedZero: carbon analytics for health care, by health care, at scale. *Lancet*. (2026) 407:205–207. [doi: 10.1016/S0140-6736(25)02280-9](https://doi.org/10.1016/S0140-6736(25)02280-9).

11. Mariette J, Blanchard O, Berné O, Aumont O, Carrey J, Ligozat AL, et al. An open-source tool to assess the carbon footprint of research. *Environ Res Infrastruct Sustain*. (2022) 2:035008. [doi: 10.1088/2634-4505/ac84a4](https://doi.org/10.1088/2634-4505/ac84a4).

12. Winter N, Marchand R, Lehmann C, Nehlin L, Trapannone R, Rokvić D, et al. The paradox of the life sciences: How to address climate change in the lab. *EMBO Rep*. (2023) 24:e56683. [doi: 10.15252/embr.202256683](https://doi.org/10.15252/embr.202256683).

13. Singh H, Weisz U, Andrew T, Blom IM, Kittipongvises S, Nansai K, et al. The Lancet Commission on Sustainable Health Care measurement framework for advancing sustainable health care transformation. *Lancet Planet Health*. (2026) 10:101398. [doi: 10.1016/j.lanplh.2025.101398](https://doi.org/10.1016/j.lanplh.2025.101398).
